# Effects of cardiovascular medications on primary patency of hemodialysis arteriovenous fistula

**DOI:** 10.1038/s41598-020-69019-6

**Published:** 2020-07-22

**Authors:** Te-I. Chang, Cheng-Hsien Chen, Hui-Ling Hsieh, Chun-You Chen, Shih-Chang Hsu, Ho-Shun Cheng, Wen-Cheng Huang, Yuh-Mou Sue, Yung-Ho Hsu, Feng-Yen Lin, Chun-Ming Shih, Shing-Jong Lin, Po-Hsun Huang, Chung-Te Liu

**Affiliations:** 1 Department of Surgery, School of Medicine, College of Medicine, Taipei Medical University, Taipei, Taiwan; 2 Division of Cardiovascular Surgery, Department of Surgery, Wan Fang Hospital, Taipei Medical University, Taipei, Taiwan; 3 Graduate Institute of Biomedical Electronics and Bioinformatics, National Taiwan University, Taipei, Taiwan; 4 Division of Nephrology, Department of Internal Medicine, Wan Fang Hospital, Taipei Medical University, Taipei, Taiwan; 5 Division of Nephrology, Department of Internal Medicine, Shuang Ho Hospital, Taipei Medical University, New Taipei City, Taiwan; 6 Department of Internal Medicine, School of Medicine, College of Medicine, Taipei Medical University, Taipei, Taiwan; 7 TMU Research Center of Urology and Kidney, Taipei, Taiwan; 8 Graduate Institute of Medical Science, National Defense Medical Center, Taipei, Taiwan; 9 Department of Radiation Oncology, Wan Fang Hospital, Taipei Medical University, Taipei, Taiwan; 10 Emergency Department, Department of Emergency and Critical Medicine, Wan Fang Hospital, Taipei Medical University, Taipei, Taiwan; 11 Department of Emergency Medicine, School of Medicine, College of Medicine, Taipei Medical University, Taipei, Taiwan; 12 Division of Cardiology, Department of Internal Medicine, Wan Fang Hospital, Taipei Medical University, Taipei, Taiwan; 13 Graduate Institute of Clinical Medicine, College of Medicine, Taipei Medical University, Taipei, Taiwan; 14 Division of Cardiology and Cardiovascular Research Center, Department of Internal Medicine, Taipei Medical University Hospital, Taipei, Taiwan; 15 Division of Cardiology, Department of Medicine, Taipei Veterans General Hospital, Taipei, Taiwan; 16 Cardiovascular Research Center, National Yang-Ming University, Taipei, Taiwan; 17 Department of Medical Research, Taipei Veterans General Hospital, Taipei, Taiwan; 18 Institute of Clinical Medicine, National Yang-Ming University, Taipei, Taiwan; 19 Taipei Medical University, Taipei, Taiwan; 20 Division of Cardiology, Heart Center, Cheng-Hsin General Hospital, Taipei, Taiwan

**Keywords:** Outcomes research, Haemodialysis

## Abstract

While the patency of vascular access is essential for hemodialysis patients, optimal pharmaceutical treatment to maintain arteriovenous fistula (AVF) patency remains lacking. As cardiovascular diseases are highly prevalent in patients with end-stage renal disease, various cardiovascular medications have also been used to maintain AVF patency. However, previous studies revealed inconsistent therapeutic effects and a comprehensive evaluation of this issue is needed. The present retrospective, longitudinal cohort study included patients receiving successful AVF creation. The evaluated cardiovascular medications included antiplatelet agents, antihypertensive agents, nitrates and nitrites, statins, dipyridamole, and pentoxifylline. The outcome was AVF primary patency. All laboratory data and medication profiles were recorded at baseline and followed at 3-month interval, until the end of the 2-year study period. Cox proportional regression model with time-dependent covariates was used to evaluate the risk for AVF patency loss. A total of 349 patients were included in the present study, in which 57% were men and the mean age was 65 ± 14 years. Among the included patients, 40% used antiplatelet agents, 27% used dipyridamole and 36% used statins at baseline. Of all the evaluated cardiovascular medications, only dipyridamole showed significant association with a higher risk for loss of AVF patency. To evaluate the effect of combination of antiplatelet agents and dipyridamole, the patients were classified into four groups, I: combine use of antiplatelet agents and dipyridamole, II: antiplatelet only, III: dipyridamole only; IV: none of both were used. Of the four groups, group IV exhibited highest AVF patency (52.4%), which was followed by group III (42.7%), group II (40%), and group I (28.6%), respectively. Compared with group IV, only group I showed a significantly higher risk for AVF patency loss. None of the cardiovascular medications evaluated in the present study showed a beneficial effect on AVF patency. Furthermore, dipyridamole showed an association with a higher risk of AVF patency loss. We do not suggest a beneficial effect of dipyridamole on maintaining AVF patency, particularly in combination with antiplatelet agents.

## Introduction

Despite various preventive measures, vascular access dysfunction remains the leading type of medical procedures and the third leading cause of hospitalization in patients undergoing maintenance hemodialysis^1,2^. Among all types of vascular access, arteriovenous fistula (AVF) shows superior long-term patency and lower infection rates compared to arteriovenous graft (AVG) or central venous catheters^3^. Therefore, AVF remains the preferred hemodialysis vascular access to date. However, AVF is not an ideal vascular access without any drawback. Among successfully created AVF, the primary patency rates range only 50–70% and 30–40% at 1 and 2 years, respectively^4,5^. The suboptimal patency rates indicate that the preventive treatments to maintain AVF patency still need to be improved.

For patients with end-stage renal disease, cardiovascular diseases are highly prevalent, including hypertension, stroke, coronary artery disease (CAD), and congestive heart failure (CHF). Thus, cardiovascular medications are frequently prescribed in this population. On the other hand, cardiovascular medications are also the mainstay of pharmaceutical treatment to maintain AVF patency^6^. Several previous studies had shown that various antiplatelet agents reduced the risk of AVF thrombosis^7–11^. In addition, several studies had shown protective effects of statins^12^, renin–angiotensin–aldosterone system blockers^13^, and calcium channel blockers^14,15^ on AVF patency. Compared with surgical or interventional measures, pharmaceutical treatment may be delivered more conveniently via oral route and seems an appealing way to maintain AVF patency.

While some studies had reported the beneficial effects of various cardiovascular medications on maintaining AVF patency^16^, many other studies showed negative results of these agents^14,17–20^, suggesting that controversy still exists on this issue. Possible explanations for these inconsistent results may be the different study designs or inadequate control of all relevant cardiovascular drugs. For example, a study focused on the effect of antiplatelet agents may not strictly control the use of angiotensin II receptor blockers or β-blockers, which may confound the conclusion. As such, a study with a thorough evaluation of various cardiovascular medications is needed to clarify this issue. Therefore, we conducted a retrospective, longitudinal cohort study to comprehensively investigate the effect of cardiovascular medications on the patency of newly created AVF.

## Methods

### Study design and subjects

The present study was conducted at Wan Fang Hospital, Taipei Medical University. Patients with newly created autologous AVF between January 2004 and May 2018 were included into the study, regardless of the site of surgery or the state of dialysis (incident hemodialysis or maintenance dialysis). The exclusion criteria included age < 20 years old, and AVF maturation failure, which was defined as AVF failed to be punctured for hemodialysis within 2 months from its creation. The study period for each patient started at the time of AVF creation, which was defined as the baseline. Each AVF patency was followed for 2 years from the time when AVF was created, and those with maintained primary patency and less than 2 years of follow-up were censored. This study was approved by the ethics committee, Institutional Review Board of Taipei Medical University (N201902034), and the informed consent was waived. The present study was conducted in accordance with the tenets of the 1975 Declaration of Helsinki, as revised in 2000.

### Guidelines fulfilment program

In the present study, the creation and surveillance of vascular access were based on “European Best Practice Guidelines on Vascular Access” as following: (1) patients were referred to nephrologist and surgeon for preparing vascular access at stage 4 of CKD to start hemodialysis with functioning vascular access. (2) Pre-operative evaluation included an ultrasound of upper extremities arteries and veins. (3) Autologous AVF was preferred over AVG and AVG was preferred over central venous catheter; the distal upper extremity AVF is preferred over proximal AVFs. (4) Objective monitoring of access function was performed weekly^21^.

### Covariates and outcomes

The demographic data, including age, gender, and comorbidities, were recorded at the time point before AVF creation, which were defined as baseline data in the present study. The comorbidities of hospitalized patients were based on the diagnosis documented in the discharge summary. For those who received AVF creation at outpatient department, the comorbidities were based on the diagnosis in outpatient medical records. The prescription of cardiovascular medications and laboratory data were recorded at baseline and every three months throughout the 2-year study period, in order to suffice time-dependent covariates for Cox regression analysis. The cardiovascular medications were classified as following categories: angiotensin inhibitors (angiotensin I converting enzyme inhibitors and angiotensin II receptor blockers), antiplatelet agents (aspirin, clopidogrel, ticlopidine), dipyridamole, dihydropyridine calcium channel blockers (CCB), non-dihydropyridine CCB, hydralazine, fibrates, nicorandil, nitrites and nitrates, pentoxifylline, statins, α-blockers, β-blockers. These medications were prescribed by attending physicians for indications approved by Taiwan Food and Drug Administration, rather than for AVF patency. In the present study, the laboratory data were measured every 1–3 months according to the requirement made by the Taiwan Society of Nephrology. The missing values were imputed by the last observation carried forward method.

The outcome of the present study was primary AVF patency, which was defined as successful use of AVF for hemodialysis without any interventional procedures^22,23^. The implementation of the first interventional procedures, including percutaneous transluminal angioplasty or surgical thrombectomy after AVF creation were defined as the end of primary patency. The outcomes were censored at the end of the 2-year study period or the time when the patients were lost from follow-up.

### Statistical analysis

Continuous variables with normal distribution were expressed as mean ± standard deviation, and continuous variable deviated from normal distribution were expressed as medians (interquartile range). The categorical variables were reported as frequencies and percentages. For two groups of continuous variables with normal distribution, Student’s t-test was used; for two groups of continuous variables deviated from normal distribution, Wilcoxon–Mann–Whitney two-sample tests was used. For more than two groups of continuous variables with normal distribution, ANOVA method was used; for more than two groups of continuous variables deviated from normal distribution, Kruskal–Wallis test was used. Normality of data distribution was tested by using Kolmogorov–Smirnov test, in which P value > 0.05 indicated normal distribution. The statistical tests for categorical variables were performed using chi-square test or Fisher’s test, as appropriate. To evaluate the association between covariates and primary AVF patency, univariate Cox proportional regression was used initially. Covariates with P-value < 0.05 in univariate Cox proportional regression were included into multivariate Cox proportional regression for primary AVF patency. The risk for primary AVF patency was expressed as hazard ratio with 95% confidence interval (HR, 95% CI). To evaluate P for trend, Cochran-Armitage test was used. Kaplan–Meier curve was used to compare the AVF patency of antiplatelet and dipyridamole users. In analysis involving medication groups, only patients who continued antiplatelet or dipyridamole use throughout the entire follow-up period were consider medication users. Statistical analysis was performed using SAS 9.4 (SAS Institute Inc., Cary, NC, USA) and Sigma Plot 10.0 (Systat Software, San Jose, CA) for Windows.

## Results

### Baseline demographic and medication profiles

The present study included 349 newly created AVFs that successfully matured for hemodialysis. The mean age of the participants was 65.2 ± 13.6 years old and 57.0% were of male gender. Overall, 44.5% had diabetes mellitus (DM), 79.0% had hypertension, 28.2% had CAD, 18.1% had dyslipidemia, 9.5% had stroke and 17.5% had CHF. Of all included AVFs, the median patency duration was 303 days. Mean serum creatinine level was 7.3 ± 4.1 mg/dL, albumin level was 3.6 ± 0.6 g/dL, calcium level was 8.3 ± 1.0 mg/dL, phosphorus level was 5.4 ± 2.0 mg/dL, glucose level was 125.0 ± 52.7. Medium serum parathyroid hormone was 175 pg/mL (Supplementary Table S1). Baseline medication profile showed that 49% used angiotensin inhibitors, 40% used antiplatelet agents, 27% used dipyridamole, 36% used statins, 49% used dihydropyridine CCB, 27% used nitrates or nitrites, 25% used α-blockers and 49% used β-blockers. (Supplementary Table S2).

### The effect of cardiovascular medications on AVF patency

To build the multivariate regression model for the risk of AVF patency, all demographic, and laboratory covariates were evaluated by univariate Cox proportional regression model. As stated previously, covariates with P values < 0.05 were considered significant to be included in a multivariate regression model. Of all the evaluated covariates, only hemoglobin and total bilirubin level showed the significance to be included into the multivariate regression model (Table 1). All classes of medications were also tested for the association with primary AVF patency by using univariate Cox proportional regression model. Of all the evaluated cardiovascular medications, only use of dipyridamole and hydralazine showed the significance to be included into the multivariate regression model. Angiotensin inhibitors, antiplatelet agents, statins, pentoxifylline, CCBs, nitrates and nitrites, nicorandil, α-blockers, and β-blockers showed no association with AVF patency in univariate analysis and were not included into multivariate analysis (Table 2).

**Table 1:** Risk factors for loss of primary patency in created AVF.

Character	HR	95% CI	P value
Male	0.85	0.64–1.13	0.252
Age: per 10 years increment	1.10	0.99–1.23	0.074
DM	1.32	0.99–1.76	0.055
HTN	1.12	0.79–1.59	0.535
CAD	1.13	0.83–1.53	0.449
Stroke	1.46	0.95–2.24	0.081
CHF	1.27	0.88–1.83	0.195
Hb: per 1 g/dL increment	1.13	1.00–1.28	0.043
Alb: per 1 mg/dL increment	1.0	0.7–1.5	0.853
Na: per 10 mmol/L increment	1.0	0.6–1.6	0.964
K: per 1 mmol/L increment	0.9	0.8–1.1	0.424
PTH: per 100 pg/mL increment	0.96	0.89–1.03	0.238
Ca: per 1 mg/dL increment	1.2	0.9–1.4	0.092
P: per 1 mg/dL increment	1.0	0.9–1.1	0.501
ALP: per 100 mg/dL increment	0.9	0.8–1.2	0.786
T. Bil.: per 1 mg/dL increment	0.41	0.17–0.97	0.043
AST: per 10 U/L increment	1.0	0.9–1.1	0.973
ALT: per 10 U/L increment	1.0	0.9–1.1	0.939
T. Chol.: per 100 mg/dL increment	0.9	0.7–1.4	0.854
TG: per 100 mg/dL increment	1.1	0.9–1.2	0.262
Ferritin: per 100 mg/mL increment	0.99	0.95–1.03	0.717
TSAT: per 10% increment	1.02	0.90–1.15	0.784

By univariate Cox proportional regression.

*AVF* arteriovenous fistula, *DM* diabetes mellitus, *HTN* hypertension, *CAD* coronary artery disease, *CHF* congestive heart failure, Hb hemoglobin, *Alb* albumin, *Na* sodium, *K* potassium, *PTH* parathyroid hormone, *Ca* calcium, *P* phosphorus, *ALP* alkaline phosphatase, *T. Bil* total bilirubin, *AST* aspartate aminotransferase, *ALT* alanine aminotransferase, *T. Chol.* total cholesterol, *TG* triglyceride, *TSAT* transferrin saturation.

**Table 2:** Cardiovascular medications and the risk for loss of primary patency in created AVF.

Character	HR	95% CI	P value
Angiotensin inhibitors	1.17	0.80–1.70	0.432
Antiplatelet agents	1.29	0.92–1.82	0.146
Dipyridamole	2.15	1.47–3.16	< 0.001
Dihydropyridine CCB	1.04	0.76–1.41	0.824
Non-dihydropyridine CCB	1.68	0.91–3.09	0.097
Hydralazine	0.31	0.10–0.97	0.044
Fibrates	1.10	0.41–2.97	0.856
Nicorandil	1.40	0.83–2.38	0.208
Nitrates and nitrites	1.16	0.77–1.7	0.469
Pentoxifylline	1.68	0.99–2.84	0.056
Statins	1.41	0.97–2.04	0.072
α-blockers	1.08	0.69–1.68	0.744
β-blockers	1.40	0.83–1.56	0.416

By univariate Cox proportional regression.

*AVF* arteriovenous fistula, *CCB* calcium channel blocker.

As a result, a multivariate Cox proportional regression model consisted of four variables of hemoglobin, total bilirubin level, use dipyridamole and hydralazine. In this multivariate regression model, only use of dipyridamole and serum total bilirubin level were significantly associated with AVF patency loss (Supplementary Table S3). As a result, the final multivariate regression model included only use of dipyridamole and serum total bilirubin level, which showed that use of dipyridamole showed a significantly higher risk for AVF patency loss. On the contrary, serum total bilirubin level was associated with a significantly reduced risk for AVF patency loss (Table 3).

**Table 3:** Risk for loss of primary patency in created AVF by multivariate Cox proportional regression.

Characters	HR	95% CI	P value
T. Bil.: per 1 mg/dL increment	0.37	0.15–0.91	0.030
Dipyridamole	2.46	1.64–3.68	< 0.001

*AVF* arteriovenous fistula, *Hb* hemoglobin, *T. Bil.* total bilirubin.

### The combination of dipyridamole and antiplatelet agents and AVF primary patency

Combination of dipyridamole and aspirin had been used in a trial to prevent thrombosis of the AVG^22^. In the following analysis, we aimed to investigate the combined effects of dipyridamole and antiplatelet agents on AVF primary patency. The participants were classified into four medication groups according to use of dipyridamole and antiplatelet agents as follows: group I, combination of antiplatelet and dipyridamole; group II: dipyridamole alone; group III: antiplatelet alone; group IV: none of both were used. Among the four medication groups, gender and age were not significantly different. Group I had significantly more CAD, while group III had more DM and CHF. Of the four medication groups, group IV exhibited highest AVF patency (52.4%), which was followed by group III (42.7%), group II (40.0%), and group I (28.6%), respectively (Table 4).

**Table 4:** Demographics of patients receiving AVF creation stratified by use of antiplatelet agents and dipyridamole.

Characters	I Combined	II Dipyridamole	III Antiplatelet	IV none	P value
Number	70	25	68	186	n/a
Male	42 (60.0%)	14 (56.0%)	40 (58.8%)	103 (55.4%)	0.906
Age	67.0 ± 13.4	69.0 ± 10.9	64.4 ± 13.4	64.3 ± 14.1	0.236
DM	44 (62.9%)	16 (64.0%)	47 (69.1%)	49 (26.3%)	< 0.001
HTN	59 (84.3%)	20 (80.0%)	57 (83.8%)	140 (75.3%)	0.294
CAD	27 (38.6%)	4 (16.0%)	26 (38.2%)	41 (22.0%)	0.006
Stroke	7 (10.0%)	4 (16.0%)	8 (11.8%)	14 (7.5%)	0.409
CHF	17 (24.3%)	2 (8.0%)	24 (35.3%)	18 (9.7%)	< 0.001
Hemoglobin (g/dL)	10.0 ± 1.9	9.7 ± 1.9	9.7 ± 1.9	9.3 ± 1.6	0.052
T. Bil	0.7 ± 0.4	0.5 ± 0.3	0.6 ± 0.5	0.5 ± 0.3	0.381
2-year primary patency	20 (28.6%)	10 (40.0%)	29 (42.7%)	97 (52.4%)	< 0.001*
Patency period (days)	190.5 (259)	352 (369)	256 (548)	480.5 (562)	0.002^†^

Statistical test by ANOVA.

*AVF* arteriovenous fistula, *DM* diabetes mellitus, *HTN* hypertension, *CAD* coronary artery disease, *CHF* congestive heart failure, *T.Bil.* total bilirubin.

*P for trend by Cochrane-Armitage trend test.

^†^Statistical test by Kruskal–Wallis test.

**Figure 1:**
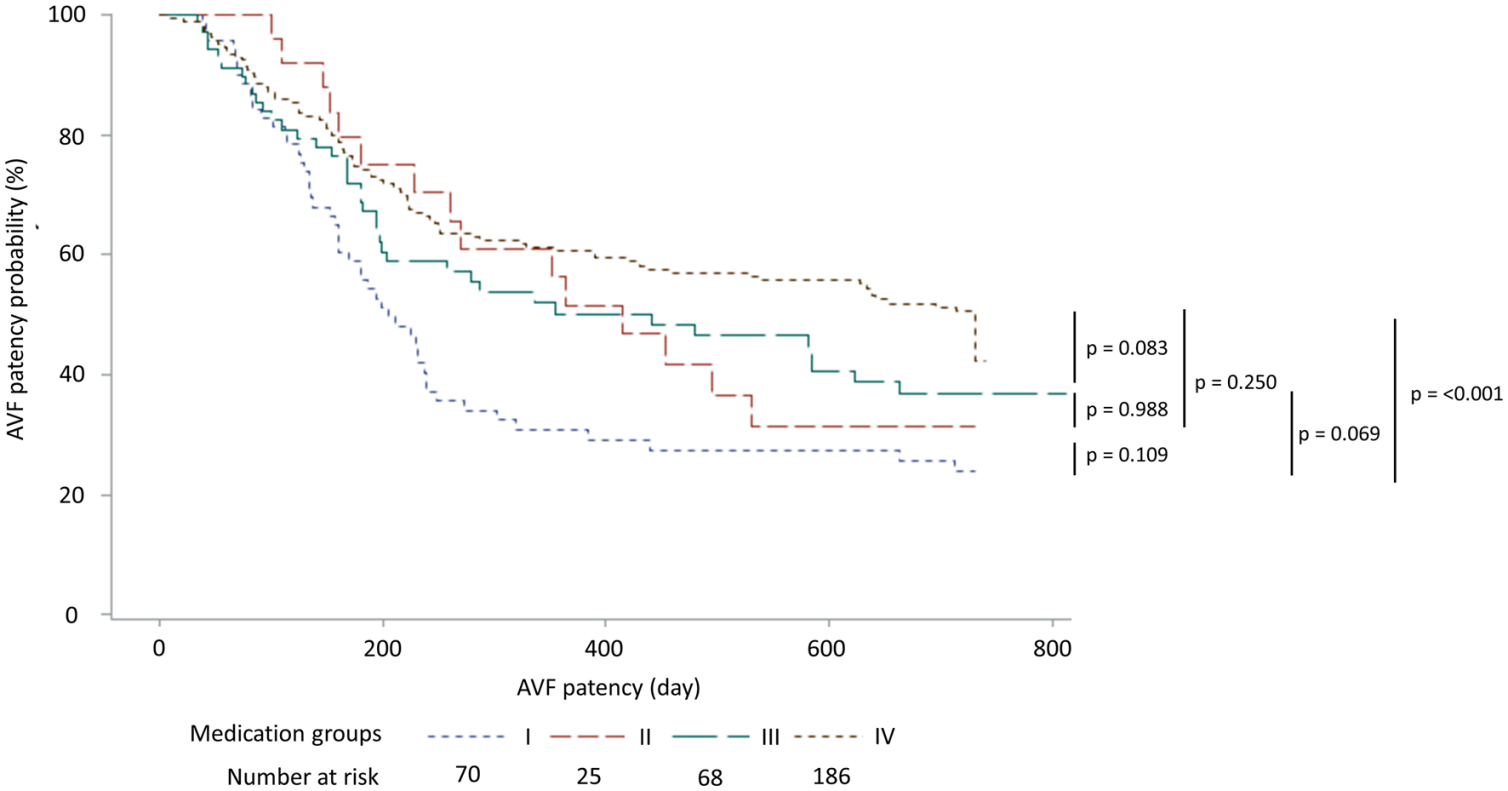
The risk for 2-year AVF patency loss with respect to the use of antiplatelet and dipyridamole. Statistical testing by Kaplan–Meier survival analysis and log rank test. Medication groups: *I* combination of dipyridamole and antiplatelets, *II* dipyridamole alone, *III* antiplatelet alone, *IV* none of both were used.

The primary patency of the four mediation groups were compared by Kaplan–Meier curve. Overall, the group I showed the lowest patency. Log-rank test showed that AVF patency in group I was only significantly lower than that in group IV. The patency between other groups were not significantly different (Fig. 1). The risk for AVF patency loss of the medication groups was further evaluated by multivariate Cox proportional regression model adjusted by the status of hemoglobin, serum total bilirubin, and use of hydralazine. In this multivariate regression model, hemoglobin, serum total bilirubin, and use of hydralazine were not significantly associated with risk of patency loss (Supplementary Table S4). As a result, the final Cox proportional regression model included only medication groups. Using group IV as the reference group, only group I showed significantly higher risk for AVF patency loss, suggesting that combined use of dipyridamole and antiplatelet agents were associated with higher risk of patency loss (Table 5).

**Table 5:** The effect of antiplatelet agents and dipyridamole on the risk for loss of primary patency in newly created AVF.

Character	Hazard ratio	95% CI	P value
**Medication groups (reference: IV)**			
I	2.10	1.48–2.98	< 0.001
II	1.38	0.80–2.38	0.254
III	1.40	0.96–2.04	0.081

Medication groups: *I* combination of dipyridamole and antiplatelets, *II* dipyridamole alone, *III* antiplatelet alone, *IV* none of both were used.

## Discussion

The findings of the present study showed that none of the evaluated cardiovascular medications were associated with improved AVF patency. In contrast, patients on dipyridamole were even at higher risk to lose AVF patency. In addition, the present study also showed that patients on combination use of antiplatelet agents and dipyridamole were at higher risk for AVF patency loss, compared to patients who were not on these medications. Based on the retrospective design of the present study, it is difficult to conclude that dipyridamole or antiplatelet agents have a harmful effect on AVF patency. However, it is difficult to suggest beneficial effects of these medications when used combined.

Neointimal hyperplasia was believed to play a major role in the pathogenesis of AVF stenosis^24^. Nonetheless, several recent studies showed that pre-existing venous neointimal hyperplasia is not associated with AVF stenosis nor maturation failure^25,26^. Furthermore, even postoperative neointimal hyperplasia in AVF wall was not associated with AVF outcomes^27^. The findings of these studies suggested that neointimal hyperplasia itself does not directly contribute to AVF failure. On the other hand, Vazquez-Padron’s group showed that inflammation of vascular smooth muscle cells and increased postoperative medial fibrosis are the main mechanism of poor AVF outward remodeling, which in turn leads to AVF stenosis and failure^28,29^. Based on the result of these studies, thrombosis is more likely a result of AVF stenosis rather than the inciting event of AVF failure. This may explain why antiplatelet agents and other cardiovascular medications play a minor role in AVF dysfunction.

Different study periods may also lead to discrepant results between studies investigating the effect of cardiovascular medications on AVF patency. In most of the randomized control trials regarding the antiplatelet effect on AVF patency, the treatment duration was 4–6 weeks, and the outcome was usually defined as AVF thrombosis at 4–8 weeks^7–11,18^. Compared to these clinical trials, the result of the present study was 2-year primary patency, which is more relevant to long-term AVF patency and dialysis adequacy. Furthermore, the medications were considered as time-dependent covariates throughout the entire study period, which exhibited more integrated medication effects on long-term AVF patency.

The present study contained 45% of patients with DM and 79% patients with hypertension, which was similar with the hemodialysis population reported in several previous studies^30,31^. In these studies, DM had been shown to be associated with a higher risk of AVF failure, which may be explained by increased thrombogenicity due to hyperglycemia^5,31^. On the contrary, another study showed that diabetes status was not associated with long-term AVF patency, which was in accordance with the findings in our study^32^. Considering these inconsistent findings and that diabetic vasculopathy is largely caused by atherosclerosis, the association between DM and AVF patency remained to be confirmed.

Medications interrupting angiotensin-aldosterone axis affecting vascular remodeling may have a beneficial effect on AVF patency. This hypothesis was tested in the Hemodialysis Fistula Maturation Study, which included 602 patients on hemodialysis via AVF. However, the study showed that angiotensin I converting enzyme inhibitor and angiotensin II receptor blocker were not associated with improved AVF patency^33^. Consistent with this study, our findings also showed that angiotensin inhibitors were not associated with AVF patency. This indicates that angiotensin or aldosterone may not be involved in the pathogenesis of neointimal hyperplasia, which remains to be investigated.

Two previous studies indicated that among all statins, atorvastatin exhibited more significant protective effect on AVF patency, suggesting that the protective effect of statin on AVF patency may be drug-specific rather than a class effect^34,35^. In the present study, all statins were considered equally in statistical analysis, which may obscure the individual effects of different statins and cause insignificant association between use of statins and AVF patency.

In the past, combination of dipyridamole and antiplatelet agents had been used in trials on hemodialysis graft patency, which showed inconsistent results^36,37^. In previous studies, dipyridamole had been reported to affect circulatory system through its antiplatelet and vasodilator properties^38^. In vitro studies showed the inhibitory effect of dipyridamole on myofibroblast transition and vascular smooth muscle cell proliferation^39,40^. Nonetheless, as these effects has not been demonstrated in vivo. One of the main findings of the present study was that combined use of dipyridamole and antiplatelet agents was associated reduced primary patency of AVF. Without an evidence from basic research, it would be impetuous to conclude an adverse effect of this combination regimen on AVF patency. However, based on the findings of our study, we do not suggest a beneficial effect of dipyridamole and antiplatelet agents on AVF patency.

As medical adjuvant treatment was not included in the present guidelines for AVF management, our findings may not cause a change to current guidelines. Nonetheless, in many hemodialysis units throughout Taiwan, antiplatelet agents and dipyridamole were commonly used for maintaining AVF patency and some patients were often convinced in its effect for this purpose. Based on the controversial effects of clopidogrel and cardiovascular medications of other classes^41^, the authors thought that these practices may not always be appropriate. Thus, the findings of the present study may help clinicians to make prescription more precisely and to avoid unnecessary medication use.

The limitations of the present study included a retrospective design that was unable to control all confounding factors. In addition, this study had a relatively small sample size that lowered the statistical power. In analysis involving medication groups, only patients who continued the respective medication use throughout the entire follow-up period were considered as medication users. This could have underestimated part of medication effects. The strength of our study included comprehensive laboratory and medication records for analysis, an optimal statistical method for long-term survival analysis, and relatively long follow-up period.

In conclusion, the findings of the present study showed that cardiovascular medications, regarding antiplatelet agents, dipyridamole, pentoxifylline, statins, dihydropyridine and non-dihydropyridine CCBs, hydralazine, and angiotensin inhibitors, did not show a beneficial effect on long-term AVF patency. In our study, dipyridamole even showed a significant association with a higher risk of AVF patency loss, in particular when combined with antiplatelet agents. Based on the retrospective design, we are not able to conclude that dipyridamole is harmful to AVF patency. However, we do not suggest their beneficial effect on maintaining AVF patency.

## Supplementary information

Supplementary Information
