# Supplementary material for: Effects of cardiovascular medications on primary patency of hemodialysis arteriovenous fistula
Source: Sci Rep. 2020 Jul 22;10:12135. doi: 10.1038/s41598-020-69019-6 (PMC7376157; doi:10.1038/s41598-020-69019-6)
Supplement: Supplementary file 1 — Supplementary Information [file 41598_2020_69019_MOESM1_ESM.docx]

**Effects of cardiovascular medications on primary patency of hemodialysis arteriovenous fistula**

Te-I Chang, MD, Cheng-Hsien Chen, PhD, Hui-ling Hsieh, MSN, Chun-You Chen, MD, Shih-Chang Hsu, MS, MD, Ho-Shun Cheng, MD, Wen-Cheng Huang, MD, Yuh-Mou Sue, MD, [Yung-Ho Hsu](https://www.sciencedirect.com/science/article/abs/pii/S0378874118309140?via%3Dihub#!), Feng-Yen Lin, PhD, Chun-Ming Shih, MD, PhD, Shing-Jong Lin, MD, PhD, Po-Hsun Huang, MD, PhD, Chung-Te Liu, MD

| **Supplemental table 1. Baseline characteristics of patients receiving AVF creation** | |
| --- | --- |
| **Characters** | Values |
| **Number, n** | 349 |
| **Male, n (%)** | 199 (57%) |
| **Age, year** | 65±14 |
| **DM, n (%)** | 155 (45%) |
| **HTN, n (%)** | 275 (79%) |
| **CAD, n (%)** | 98 (28%) |
| **Dyslipidemia, n (%)** | 63 (181%) |
| **Stroke, n (%)** | 33 (10%) |
| **CHF, n (%)** | 61 (18%) |
| **Patency period, day** | 303 (576) |
| **Hemoglobin, g/dL** | 9.6±1.8 |
| **BUN, mg/dL** | 74.6±38.4 |
| **Cr, mg/dL** | 7.3±4.1 |
| **Albumin, g/dL** | 3.6±0.6 |
| **Na, mmol/L** | 137.2±3.9 |
| **K, mmol/L** | 4.5±0.8 |
| **Ca, mg/dL** | 8.3±1.0 |
| **P, mg/dL** | 5.4±2.0 |
| **PTH, pg/mL** | 175 (281) |
| **AST, U/L** | 22 (12) |
| **ALT, U/L** | 17 (10) |
| **ALP, mg/dL** | 74 (36.5) |
| **T. Chol., mg/dL** | 184.3±51.1 |
| **Ferritin, ng/mL** | 260.4 (335.8) |
| **TSAT, %** | 21.7 (13.6) |
| **Glucose, mg/dL** | 125.0±52.7 |

AVF, arteriovenous fistula; DM, diabetes mellitus; HTN, hypertension; CAD, coronary artery disease; CHF, congestive heart failure; BUN, Blood urea nitrogen; Cr, creatinine; Na, sodium; K, potassium; Ca, calcium; P, phosphorus; PTH, parathyroid hormone; AST, aspartate aminotransferase; ALT, alanine aminotransferase; ALP, alkaline phosphatase; T. Chol., Total cholesterol; TSAT, transferrin saturation.

| **Supplemental table 2. Baseline cardiovascular medications of patients receiving AVF creation** | |
| --- | --- |
| **Medication** | **Number (%)** |
| **Angiotensin inhibitors** | 170 (49%) |
| **Antiplatelet agents** | 138 (40%) |
| **Dipyridamole** | 95 (27%) |
| **Dihydropyridine CCB** | 172 (49%) |
| **Non-dihydropyridine CCB** | 52 (15%) |
| **Hydralazine** | 54 (16%) |
| **Fibrates** | 37 (11%) |
| **Nicorandil** | 55 (16%) |
| **Nitrates or nitrites** | 94 (27%) |
| **Pentoxifylline** | 64 (18%) |
| **Statins** | 125 (36%) |
| **α-blockers** | 86 (25%) |
| **β-blockers** | 170 (49%) |

AVF, arteriovenous fistula; CCB, calcium channel blocker.

| **Supplemental table 3. Risk for loss of primary patency in created AVF by multivariate Cox proportional regression** | | | |
| --- | --- | --- | --- |
| **Characters** | **HR** | **95% CI** | **P value** |
| **Hb: per 1 g/dL increment** | 1.11 | 0.98-1.26 | 0.105 |
| **T. Bil.: per 1 mg/dL increment** | 0.39 | 0.16-0.96 | 0.040 |
| **Dipyridamole** | 2.30 | 1.53-3.45 | <0.001 |
| **Hydralazine** | 0.42 | 0.13-1.31 | 0.134 |

AVF, arteriovenous fistula; Hb, hemoglobin; T. Bil., total bilirubin.

| **Supplemental table 4. The effect of antiplatelet agents and dipyridamole on the risk for loss of primary patency in newly created AVF** | | | |
| --- | --- | --- | --- |
| **Character** | **Hazard ratio** | **95% CI** | **P value** |
| **Hemoglobin (per 1 increment)** | 1.11 | 0.98-1.26 | 0.110 |
| **T.Bil (per 1 increment)** | 0.48 | 0.20-1.14 | 0.094 |
| **Hydralazine use** | 0.78 | 0.50-1.23 | 0.281 |
| **Medication groups (reference: IV)** |  |  |  |
| **I** | 2.25 | 1.50-3.39 | <0.001 |
| **II** | 1.61 | 0.90-2.86 | 0.107 |
| **III** | 1.61 | 1.03-2.51 | 0.035 |

Medication groups: I, combination of dipyridamole and antiplatelets; II, dipyridamole alone; III, antiplatelet alone; IV, none of both were used.
